# Spotted Fever: Epidemiology and Vector-*Rickettsia-*Host Relationship in Rio de Janeiro State

**DOI:** 10.3389/fmicb.2017.00505

**Published:** 2017-03-30

**Authors:** Diego C. Montenegro, Karla Bitencourth, Stefan V. de Oliveira, Ana P. Borsoi, Karen M. Cardoso, Maria S. B. Sousa, Cristina Giordano-Dias, Marinete Amorim, Nicolau M. Serra-Freire, Gilberto S. Gazêta, Reginaldo P. Brazil

**Affiliations:** ^1^Laboratório de Doenças Parasitária, Instituto Oswaldo Cruz/Fundação Oswaldo CruzRio de Janeiro, Brazil; ^2^Laboratório de Referência Nacional em Vetores das Riquetsioses – Secretaria de Vigilância em Saúde/Ministério da Saúde, Instituto Oswaldo Cruz/Fundação Oswaldo CruzRio de Janeiro, Brazil; ^3^Secretaria de Vigilância em Saúde – Ministério da SaúdeBrasilia, Brazil; ^4^Secretaria de Estado de Saúde do Rio de JaneiroRio de Janeiro, Brazil

**Keywords:** public health, eco-epidemiology, rickettsioses, tick-borne diseases, zoonosis

## Abstract

The eco-epidemiological scenario of spotted fever (SF), a tick-borne disease that affects humans and other animals in several countries around the world, was analyzed in Rio de Janeiro (RJ) State, Brazil. During the last 34 years, 990 SF cases were reported in RJ (the Brazilian state with the highest population density), including 116 cases confirmed by serology (RIFI) or PCR, among 42.39% of the municipalities with reported cases of SF. The epidemiologic dynamics of SF in RJ State are very heterogeneous in time and space, with outbreaks, high mortality rates and periods of epidemiological silence (no SF cases reported). Furthermore, it exhibited a changing epidemiological profile from being rural to becoming an urban disease. This study identified arthropods infected with *Rickettsia felis*, *R. bellii* and *R. rickettsii*, and found that the abundance of ectoparasites was associated with specific hosts. The *R. rickettsii*-vector-host relationship was most evident in species-specific parasitism. This suggests that the association between dogs, cattle, horses, capybaras and their main ectoparasites, *Rhipicephalus sanguineus* and *Ctenocephalides felis*, *Rhipicephalus microplus, Dermacentor nitens*, and *Amblyomma dubitatum*, respectively, has a key role in the dynamics of *R. rickettsii* transmission in enzootic cycles and the maintenance of carrier ectoparasites, thus facilitating the existence of endemic areas with the ability to produce epidemic outbreaks of SF in RJ. This study found confirmed human infections for only the *R. rickettsii* carrier *Amblyomma sculptum*, which reinforces the importance of this species as a vector of the pathogen in Brazil. This study can be adapted to different eco-epidemiological scenarios of spotted fever throughout the Americas.

## Introduction

Rickettsioses are diseases caused by obligate intracellular bacteria of the genus *Rickettsia*. Different species of the Spotted-Fever Group of *Rickettsia* (SFGR) are considered etiologic agents of spotted fever (SF), a zoonosis widely distributed throughout the world, with seasonal and sporadic outbreaks that may involve high mortality rates (Raoult and Roux, 1997; Rudakov et al., 2003; Parola et al., 2009, 2013; Eremeeva and Dasch, 2015).

In nature, the SFGR transmission-cycle is maintained by the capacity of ticks to act as vectors, reservoirs and/or amplifiers of the bacteria. Ticks can remain infected throughout their lives through transovarial and/or transstadial transmission. The continued presence of the bacteria in a population may also be the result of ticks obtaining a blood meal, and transmitting the bacteria to a wide variety of mammals, including humans (Dumler and Steohen, 2005; Parola et al., 2013; Eremeeva and Dasch, 2015).

Spotted fever is the most prevalent tick-borne illness in Brazil (Fiol et al., 2010; Brasil, 2014a), having been first reported in the country in the early 20th century (Fialho, 1929; Piza et al., 1931). A variety of species of *Rickettsia*, and their vectors, have since been identified in areas with reported cases of SF (Cunha et al., 2009; Gehrke et al., 2009; Medeiros et al., 2011; Szabó et al., 2013, Moura-Martiniano et al., 2014), illustrating the complexity of its enzootic and epidemic cycles, as well as the diversity of potential vectors.

There have been confirmed cases of SF in 21 out of 23 Brazilian states (Brasil, 2015a). Morbidity is moderate, but the lethality rate is high, exceeding 80% in severe cases (Szabó et al., 2013; Brasil, 2014a), when the rash becomes petechial and then hemorrhagic, consisting mainly of bruises or suffusions (Brasil, 2014a).

The Southeast Region of Brazil has the greatest number of confirmed cases of, and the most deaths by, SF (Brasil, 2015a), with outbreaks in different eco-epidemiological scenarios (Medeiros et al., 2011, Szabó et al., 2013, Moura-Martiniano et al., 2014; Nasser et al., 2015). Furthermore, within this region, the geopolitical space of Rio de Janeiro (RJ) State stands out as having one of the highest population densities in the country, experiences great tourism and possesses large urban and rural areas in different ecoregions.

Although some studies on SF in the Americas have addressed a holistic view of the ecological components associated with the epidemiological dynamics of the disease (Labruna, 2009; Parola et al., 2009; Eremeeva, 2012; Quintero et al., 2013; Szabó et al., 2013), studies in endemic areas are scarce. Therefore, the present study aims to analyze the vector-*Rickettsia*-host relationship in RJ and its relation to the dynamics of SF cases from the 1980s to 2014, in order to assess the factors that are associated with SF in RJ.

## Materials and Methods

### Study Area

**FIGURE 1:**
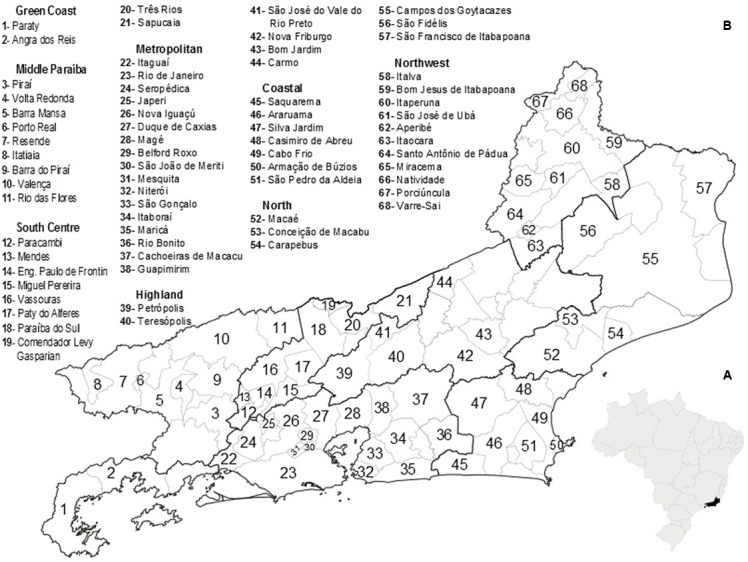
**(A)** Map of Brazil highlighting the location of Rio de Janeiro State. **(B)** Health division of Rio de Janeiro State, Brazil, indicating the municipalities that contributed to the analyzed data. Source: Secretaria de Estado de Saúde do Rio de Janeiro- http://www.saude.rj.gov.br/.

Located in the eastern portion of the Southeast Region of Brazil, RJ) occupies an area of 43,777.954 km^2^, and is comprised of 92 municipalities (**Figure 1**). It has the largest population density in Brazil (365.23 inhabitants/km^2^), with an estimated population, in 2015, of 16,550,024 inhabitants. It is the most urbanized region of the country, with 97% of the population living in cities (IBGE, 2015).

### Epidemiological Data

Epidemiological data were obtained from the Secretary of State of Health of RJ (SES/RJ) and from the Sistema de Informação de Agravos de Notificação (SINAN) (Brasil, 2015b), for the time frame from 1980 to 2014. The data were chronologically divided into two periods: Period 1 corresponding to the years from 1980 to 2004; and Period 2 corresponding to the years from 2005 to 2014. This separation of data was related to the epidemiological dynamics of reported cases. In the second period, epidemiological descriptive data (time, place and person) are included in SINAN reporting forms.

### Putative Vectors of SF

Information regarding potential vectors of SF was obtained from the database of the Laboratório de Referência Nacional em Vetores das Riquetsioses (LIRN; Labortory of the National Reference of Rickettsial Vectors), built from samples analyzed and received within the flow of the Rede Nacional de Vigilância de Ambiental para Febre Maculosa e outras Rickettsioses (National Network for Environmental Monitoring for Spotted Fever and other Rickettsial Diseases), of Ministry of Health from 2005 to 2014. The samples were collected in sampling units (specimens originating from the same host or environment, collected on the same date and during the same epidemiological outbreaks) by state and municipal health teams from RJ for environmental monitoring and investigating cases of SF.

In general, the samples were collected by cloth-dragging, visual searches on hosts and inspection of plant litter and abiotic surfaces.

Ticks were identified using dichotomous keys for larvae (Amorim and Serra-Freire, 1999), nymphs (Martins et al., 2010), and adults (Aragão and da Fonseca, 1961; Barros-Battesti et al., 2006), or specific descriptions (Maroun et al., 1999; Walker, 2000). Fleas were identified according to Linardi and Guimarães (2000), and lice according to Ferris (1951) and Price et al. (2004).

Voucher specimens were deposited in Coleção de Artrópodes Vetores Ápteros de Importância em Saúde das Comunidades – CAVAISC/FIOCRUZ, while the remaining specimens were submitted to total DNA extraction by salt-extraction technique (Aljanabi and Martinez, 1997). Species of *Rickettsia* were identified using PCR. In this process, samples were analyzed as single or pooled specimens ranging from 2 to 10 individuals of the same sampling units, stage of development (larva, nymph, and adult), and sex. For PCR, we used *Rickettsia* genus-specific primers targeting the *gltA* gene (CS4 239/CS4 1069, CS2 78/CS2 323) (Labruna et al., 2004) and SFG-specific primers targeting the *ompA* gene (*Rr* 190.70p/ *Rr* 190.602n) (Regnery et al., 1991).

Amplified DNA fragments were visualized using 2% agarose gel stained with ethidium bromide and observed using a gel scanner with ultraviolet light (Sambrook and Russell, 2001).

Previous research (Gehrke, 2010; Moura-Martiniano et al., 2014) used ectoparasites from SF research and surveillance areas in RJ, collected throughout 2005–2009. In these studies, amplicons that corresponded to the expected amplified size were purified and sequenced. The resulting sequences were identified by comparison with the GenBank database using BLAST. These sequences were also submitted to phylogenetic reconstruction. All obtained sequences were deposited in GenBank (Supplementary Table 1).

### Data Analysis

All information obtained was tabulated according to the type of origin (investigation of cases or regular environment surveillance of SF) and submitted to descriptive statistical analysis; frequency measures, in the case of epidemiological data; and analyses of the percentage of similarity – SIMPER (Clarke, 1993) for biological data. SIMPER was used to highlight species with the highest contribution to abundance among the municipalities of RJ using standardized data of richness and abundance of vectors and the association measure of Bray–Curtis using the statistical package Plymouth Routines In Multivariate Ecological Research – PRIMER-v.5 (Clarke and Gorley, 2006).

To identify statistical patterns of association between parasites (dependent variable) and hosts (independent variable), biological data determined most representative by SIMPER analyses were used for exploratory non-metric multi-dimensional scaling (NMMDS) (Clarke, 1993) using PRIMER-E. Data were standardized with an association measure of Bray–Curtis. In these analyses, the *Bubble* value of each association is graphically represented on a Cartesian plane by the size (number of individuals) and frequency (number of samples) of each species of ectoparasite collected from each host. For visual comparisons, Bubble groups of each analysis were allocated in the same plane by using ruler measurements and the line grid of the Paint program of Microsoft Office package.

Using the program TerraView (INPA, 2010), and data by area, cartographic analyses were performed using epidemiological thematic maps, with vectors and natural infection with Rickettsial genes to determine distribution, probabilistic patterns and the relationship between these variables using kernel density estimators.

## Results

### Epidemiology

**FIGURE 2:**
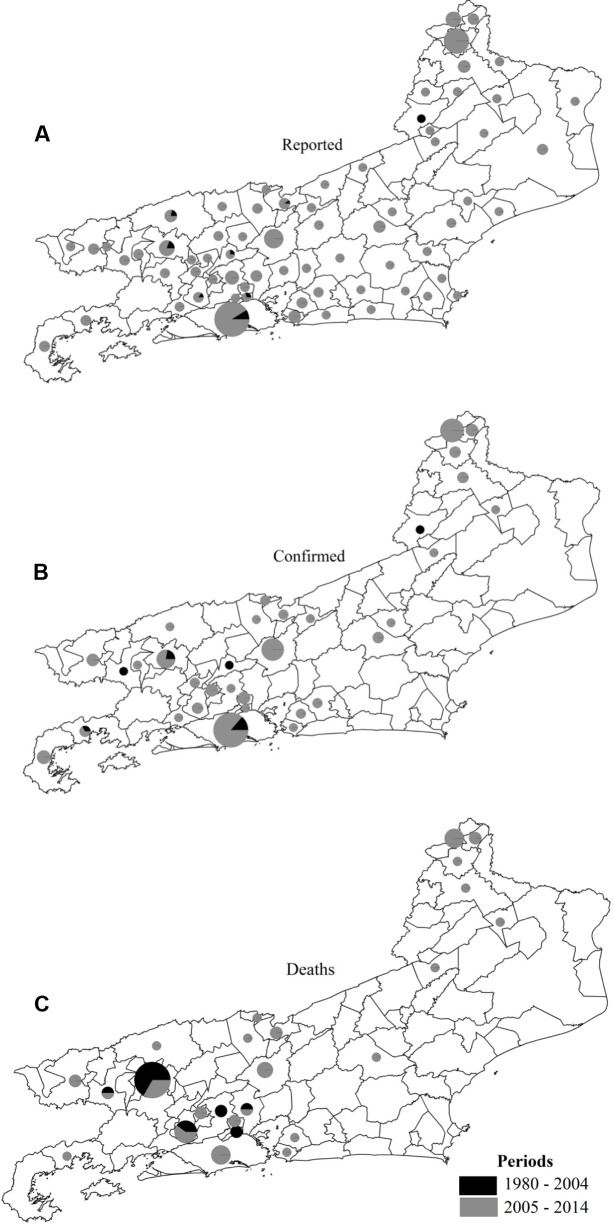
**Reported cases (A)**, confirmed cases **(B)**, and deaths **(C)** from spotted fever in municipalities of Rio de Janeiro State in two study periods.

Nine hundred and ninety suspected SF cases were reported during the study period in 73.91% (68/92) of the municipalities within the study area. Of the 990 cases, 11.71% (116/990) were confirmed, including patients residing in 42.30% (39/92) of the municipalities with reported SF cases (**Figures 2A,B**). Five municipalities (Barra do Pirai, Paraty, Petrópolis, Portiuncula, and Rio de Janeiro) possessed 52.59% (61/116) of the confirmed cases. The municipality of Rio de Janeiro, the State’s capital, had the highest rate, with 18.97% (22/116) of the cases (**Figure 2B**). In 64.10% (25/39) of the municipalities with confirmed cases, there were 48 deaths (**Figure 2C**).

**FIGURE 3:**
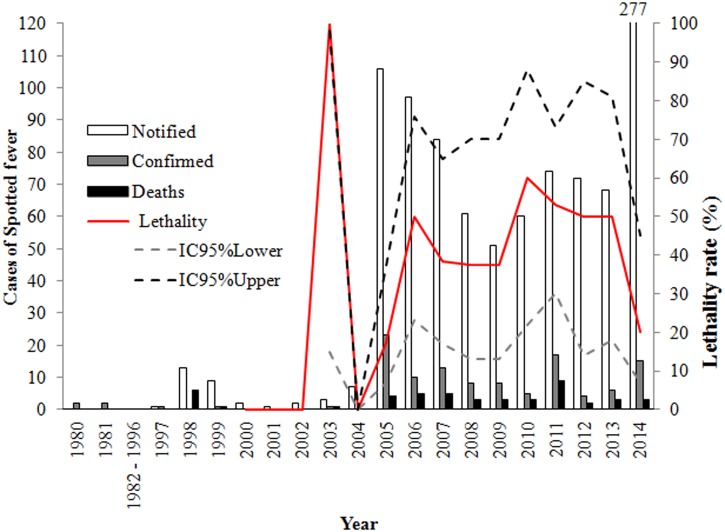
**Epidemiological dynamics of spotted fever in Rio de Janeiro State, from the 1980s to 2014**.

The epidemiological dynamics of SF in the study area shows chronologically different profiles (**Figure 3**). In the first period, between 1980 and 2004, records were mainly quantitative with little additional information. During this period, there were 41 reported cases, with 9 being confirmed and 14 reported deaths.

In the second period, between 2005 and 2014, 949 cases were reported, with 28.11% (107/949) being confirmed and 37.38% (40/107) advancing to death. Confirmed cases were distributed throughout the year, with higher frequencies during the months of July to October. The reported cases of this period also possessed a greater amount of descriptive data. From 2007 to 2014, in 28.95% (22/76) of the cases, the infection occurred in an urban area, 13.15% (10/76) in a peri-urban area and 35.53% (27/76) in a rural area, with the area of infection being unknown in 22.37% (17/76) of the cases.

Households infections occurred in 27.63% (21/76) of the cases, while 22.37% (17/76) occurred during leisure activities and 17.11% (13/76) during labor activity, while 32.89% were not specified.

Males were more frequently infected than females, accounting for 61.84% (47/76) of the infections. In terms of age, the population of reproductive age and the work force, between 20 and 59 years, was the most compromised (57.33%) age group. Even within that period (2007–2014), we found eight cases of SF caused by *R. rickettsii* (five turned to death, one progressed to healing and two ignored) and one case caused by *R. parkeri* (progressing to cure).

### Richness and Abundance of Vectors

Distributed among 650 samples, 8,064 specimens of 14 species of wingless arthropods of interest to public and animal health were identified.

Ticks (Acari: Ixodidae) had the greatest abundance and species richness. In order of contribution, the species are: *Amblyomma sculptum, Rhipicephalus sanguineus, Rhipicephalus microplus, Dermacentor nitens, Amblyomma aureolatum, Amblyomma dubitatum, Ornithodoros* spp., *Amblyomma ovale, Haemaphysalis leporispalustris* and *Amblyomma longirostre* (data not shown). The representation of Insecta was dominated by the flea *Ctenocephalides felis*, with *Polygenis* spp, *Ctenocephalides canis* occurring in very low frequency, and the lice *Felicola felis* and *Pediculus humanus* occurring in even lower frequency (data not shown).

Of the 37 municipalities with samples sent to LIRN, 30 occurred during the investigation of cases, 10 of which were also part of regular environment surveillance of SF. Seven municipalities had information only for environmental monitoring.

According to SIMPER analysis, the structure of the community of arthropod-carriers of *Rickettsia* spp. is heterogeneous in the study area, and independent of the origin of the samples (investigation or monitoring). Four species of ticks (*A. sculptum*, *Rh. sanguineus, Rh. Microplu* and *D. nitens*) and one flea (*Ct. felis*) contributed to 99% of the total arthropod-carrier abundance. However, the contribution of *A. sculptum* to total relative abundance was dominant in both foci and environmental monitoring areas with 78.67 and 48.86%, respectively.

### Vector-Host Relationship

Potential *Rickettsia* vectors were found parasitizing eight mammalian species, *Bos taurus* (cattle), *Canis familiaris* (dog), *Capra hircus* (sheep), *Equus asinus* (ass) *Equus caballus* (horse), *Felis catus* (cat), *Hidrochoerus hidrochaeris* (capybara) and *Homo sapiens* (human); and two morphospecies Mula and *Bos* spp. In the environment, mainly immature stages (larvae and nymphs) of *A. sculptum* and *Rh. microplus* were found.

**FIGURE 4:**
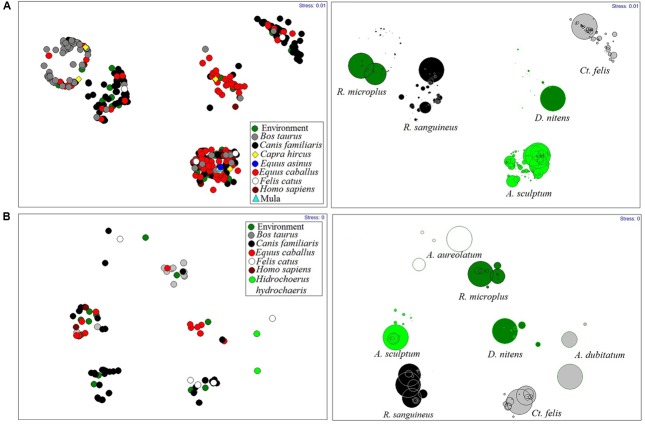
**Rickettsial vectors modeled by their hosts in Rio de Janeiro State, from 2005 to 2014. (A)** During investigation of spotted fever cases, **(B)** In environment surveillance.

Using the five species that contributed 99% of the total abundance, the NMMDS analyses, with stress values in 2D and 3D of 0, found no statistically significant spatial distribution model for vectors in municipalities. With the same amount of stress, NMMDS found that the distribution of ectoparasites to be modeled by their hosts (**Figure 4**).

Except for *A. sculptum*, in areas under investigation or surveillance, most species were associated with a single host. Thus, for example, the horse is responsible for maintaining the populations of *D. nitens*, *B. taurus* modeled populations of *Rh. microplus* and the dog was the host most parasitized by *Rh. sanguineus* (**Figure 4**).

### Vector-Pathogen-Host Relationship

The presence of the *glt*A gene was detected in 26 samples of ticks and 24 samples of fleas. Yet, in 43 samples of ticks and 15 of fleas the *ompA* gene was amplified. Sequences obtained from some samples of RJ ectoparasites exhibited identities of between 97 and 100% with *R. rickettsii* and *R. felis* (**Table 1** and Supplementary Table 1). In those species in which *R. rickettsii* genes were found, the ectoparasites had been collected on their specific hosts. *A. sculptum* was the only species found parasitizing humans with the presence of genes of the causative agent of Brazilian spotted fever (**Table 1**).

**Table 1:** Vector/carrier-*Rickettsia*-host relationship in Rio de Janeiro State, 2005–2014.

Host∖Vector	*Amblyomma sculptum*	*Rhipicephalus sanguineus*	*Ctenocephalides felis*	*Ctenocephalides canis*	*Rhipicephalus microplus*	*Dermacentor nitens*	*Amblyomma* spp.	*Amblyomma aureolatum*	*Amblyomma dubitatum*
Environmental	R	R, R-GSF	R. rick, R. fe		∗	∗	∗	∗	∗
*Bos taurus*	R, R-GSF				R, R-GSF, R. rick				
*Canis familiares*	R, R-GSF, R. rick	R, R-GSF, R. rick	R, R-GSF, R. fe, R. rick	R. rick	∗	∗	R	R	
*Capra hircus*	∗				∗				
*Equus asinus*	∗								
*Equus caballus*	R, R-GSF, R. rick				R-GSF	R, R-GSF, R. rick			
*Felis catus*	∗		R, R-GSF, R. fe, R. rick					∗	∗
*Hydrochoerus hydrochaeris*									R. rick*, R. belli*
*Homo sapiens*	R-GSF, R. rick				∗		∗		
Mula						∗			

*R, Rickettsia; R-SFG, Rickettsia of Spotted fever group; R. rick, Rickettsia rickettsii; R. fe, Rickettsia felis. ∗ Infestation without detection of Rickettsia.*

**FIGURE 5:**
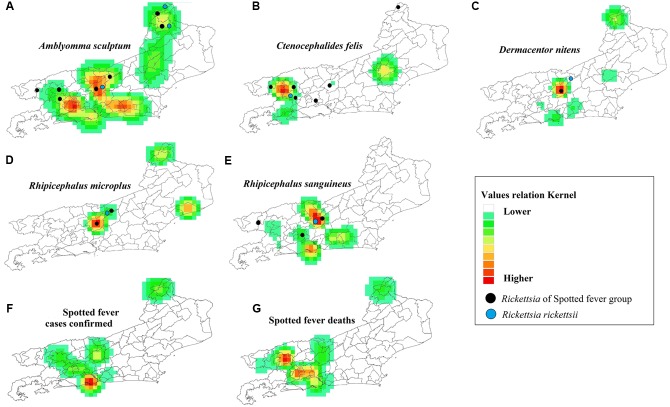
**Probabilistic maps in Rio de Janeiro State.** Occurrence of the main vectors/carrier of *Rickettsia* **(A–E)**, occurrence of cases of SF **(F)** and deaths due to SF **(G)**. Detection of *R. rickettsii* gene (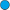) and *Rickettsia* Spotted-Fever Group (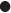).

Probabilistic analyses of the distribution of the species *A. sculptum, Rh. sanguineus* and *Ct. felis*, with the presence of genes for SFGR and *R. rickettsii*, had the same spatial model as the likelihood of cases and deaths in the SF study area (**Figure 5**). The likelihood of cases and deaths by SF was found to be higher in the municipalities of Metropolitan, Middle Paraíba and Northwest Regions of RJ (**Figure 5**).

## Discussion

The first reports of SF in the study area were in the 1940’s, and it was considered a disease of rural areas for several decades (Tostes and Bretz, 1941; Greca et al., 2008). More recently the profile of SF has changed, with cases of infection reported for urban and peri-urban areas between 2007 and 2014 outweighing cases in rural areas because of the greater population density. However, the process of SF urbanization does not seem to be common throughout Brazil (Barros-Silva et al., 2014), but it is evident in the southeastern part of the country (Nasser et al., 2015; Souza et al., 2015). Some studies have reinforced the importance of areas experiencing urban expansion and deforestation, or places where work is done with mammals in confinement, and other environmental determinants in the epidemiological dynamics of SF (Gazeta et al., 2009; Labruna, 2009; Ogrzewalska et al., 2011; Moura-Martiniano et al., 2014; Rozental et al., 2014; Nasser et al., 2015). In fact, regions of higher density seem more conducive to the emergence of cases in southeastern Brazil, even in rural areas (Ribeiro et al., 2013).

The observed epidemiological dynamics of SF overtime (**Figure 2**) are probably related to the perception of the disease in the context of varying health policies at different time periods, which seems to have influenced the sensitivity of the health system in detecting cases. Investigations into the sensitivity of the epidemiological surveillance system for the catchment, diagnosis and management of patients with SF are being prepared for publication.

Thus, between 1980 and 2000, SF was only reported for the most morbid cases and mainly during a few recognized outbreaks. As of 2001, reporting of SF cases became mandatory in Brazil (Brasil, 2001), and although there are studies that show cases and deaths in 2001 in RJ (De Lemos et al., 2002), the first confirmed cases of SF appeared in SINAN in 2003.

However, the number of cases increased significantly from 2005 due to wide media exposure of a SF outbreak in the Highland Region of RJ. Similarly, a better definition of the epidemiological profile of SF was integrated into the new notification system in 2007 (Brasil, 2009), when new variables were included in reports; in particular the evolution of cases, which allows mortality rates to be determined, and clearly defining cases with diagnostic criteria.

Beginning in 2012, with the implementation of the national network for environment surveillance of SF and other Rickettsial diseases in Brazil (de Oliveira et al., 2015), and with Ordinance N° 1.271 (Brasil, 2014b), in which SF and other Rickettsial diseases were included as diseases of immediate compulsory notification, it became evident that some municipalities of RJ actively participated in environmental surveillance and the reporting of suspicious and actual cases, resulting in an increase in the number of municipalities reporting SF and the number of overall reported SF cases (**Figures 1**, **5**).

In general, within the study area the disease is first diagnosed as leptospirosis or dengue, and when the case progresses to death, SF is suspected (Lamas et al., 2008; Moliterno, 2009; Monteiro et al., 2014; Rozental et al., 2014).

All these scenarios can be considered important contributors to the dissonant dynamics of confirmed cases and deaths of SF in RJ (**Figures 1**, **3B,C**).

Most ectoparasites, excluding *A. sculptum*, parasitize specific hosts, which seems to be common (Serra-Freire and Furlong, 1993; Gazeta et al., 2009; Gehrke et al., 2009). The obtained sequences of *R. rickettsii*, from *glt*A (*Rickettsia*) and *ompA* (SFGR) genes, are prevalent in this particular parasitism, suggesting that the association between vertebrates and their main ectoparasites plays a key role in the dynamics of *Rickettsia* transmission in enzootic cycles, which provides endemic areas with the opportunity to give rise to outbreaks of SF.

The present study shows a relationship between the spatial distribution of *A. sculptum, Rh. sanguineus* and *Ct. felis* infected with cases of SF, although some studies have also identified these species in areas with incidence of SF cases in RJ (Cunha et al., 2009; Gazeta et al., 2009; Gehrke et al., 2009; Moura-Martiniano et al., 2014). However, the first tick species (*A. sculptum*) has been previously incriminated in the transmission of *R. rickettsii* to humans in Brazil (Greca et al., 2008; Brasil, 2009, 2014a; Szabó et al., 2013).

The results of the present study also found parasitism by *A. sculptum* infected with *R. rickettsii* on humans, which confirms the importance of this species as a vector of this SF agent. Therefore, we consider the other species of ticks (*Rh. sanguineus*, *Rh. microplus*, and *D. nitens*) and the flea (*Ct. felis*) found possessing Rickettsial genes, to be of *carrier* status (Estrada-Peña et al., 2013).

Although this study is the first to spatially display SF endemic areas (**Figures 3B**, **5F**), occurrence areas of the main hosts, vectors and arthropod carriers of *R. rickettsii* and SFGR (**Figure 5**), it has some limitations that must be addressed: (1) Epidemiologically there may have several mistake that could not be standardized for a retrospective study; (2) In the routine surveillance system of *Rickettsia* vectors with SFGR genes, few samples were diagnosed to species level, which limits their contribution to molecular taxonomy of *Rickettsia*; it does not allow the determination of whether *R. parkeri* is really circulating in RJ or if it can be associated with cases of SF as data from SES/RJ indicate (one case); and (3) There are other factors that may have had an affect on our results, including the sampling effort, the different techniques of collection, the time between the occurrence of an event and its corresponding case study and the lack of information on vectors in municipalities with confirmed cases of SF.

Future research on the spatial distribution of areas of probable infection, molecular taxonomy of *Rickettsia* in the main vectors, serology of the main hosts identified in this study, relationship between tick phenology and environmental (abiotic) features, application of mathematical models to determining tick niches from survey data (Estrada-Peña et al., 2013), are needed for a better understanding of disease dynamics and vulnerability factors so as to have a more focused perspective on prevention and control by grievance.

## Author Contributions

DM contributed to the concept, design and statistical analysis of the work; MA, KB, AB, CG-D, GG, MS, and NS-F with the collection and taxonomic identification of ectoparasites; KB, GG, and KC contributed to PCR techniques for Rickettsial identification in ticks and fleas; MA, CG-D, and GG with acquisition of the data; SO, RB, KB, CG-D, GG, and DM with analysis, interpretation and the drafting the work. All contributed revising it critically for important intellectual content; final approval of the version to be published; and all are agreement to be accountable for all aspects of the work in ensuring that questions related to the accuracy or integrity of any part of the work are appropriately investigated and resolved.

## Conflict of Interest Statement

The authors declare that the research was conducted in the absence of any commercial or financial relationships that could be construed as a potential conflict of interest.
