# Supplementary material for: Spotted Fever: Epidemiology and Vector-Rickettsia-Host Relationship in Rio de Janeiro State
Source: Front Microbiol. 2017 Mar 30;8:505. doi: 10.3389/fmicb.2017.00505 (PMC5371726; doi:10.3389/fmicb.2017.00505)
Supplement: Supplementary file 1 [file Table_1.DOC]

**Supplementary Table 1.** Relationship between sequencing *Rickettsia,* host and putative vector in the municipalities of Rio de Janeiro State

| **Municipalities** | **Host** | **Species** | **Rickettsia detection** | | |
| --- | --- | --- | --- | --- | --- |
|  |  |  | **Specimen** | **Gene amplified** | **Identity sequencing (access GenBank)** |
| Petrópolis | *Bos taurus* | *Rhipicephalus microplus* | 3F | omp*A* |  |
|  |  |  | 1F | glt*A* |  |
|  |  | *Amblyomma sculptum* | 1M | glt*A* and omp*A* |  |
|  | *Free Living* | *Amblyomma sculptum* | 12N | glt*A* and omp*A* |  |
|  |  |  | 1M+ 18N | glt*A* |  |
|  |  |  | 1N + 1F | omp*A* |  |
|  |  | *Ornithodoros* sp. | 8L | glt*A* and omp*A* |  |
|  |  | *Rhipicephalus sanguineus* | 1F | glt*A* |  |
|  | *Homo sapiens* | *Amblyomma sculptum* | 1M | glt*A* and omp*A* |  |
|  |  |  | 2F+ 5N | omp*A* |  |
|  |  |  | 2N | glt*A* |  |
|  |  |  | 1N | glt*A* | 97 % *Rickettsia rickettsii*^1^ (JN400369) |
|  | *Canis familiaris* | *Amblyomma sculptum* | 1N | omp*A* |  |
|  |  | *Rhipicephalus sanguineus* | 1F | glt*A* | 100% *Rickettsia rickettsii*^1^ (JN400370) |
|  |  | *Amblyomma sp.* | 2N | glt*A* |  |
|  |  | *Amblyomma aureolatum* | 1M | glt*A* |  |
|  | *Equus caballus* | *Dermacentor nitens* | 1M | glt*A* | 100% *Rickettsia rickettsii*^1^ (JN400371) |
|  |  |  | 1F + 1M | omp*A* |  |
|  |  | *Amblyomma sculptum* | 1N+ 4M +1F | omp*A* |  |
|  |  |  | 1M | glt*A* |  |
| Porciúncula | Free Living | *Amblyomma sculptum* | 1N | omp*A* |  |
|  |  | *Amblyomma dubitatum* | 1F | glt*A* and omp*A* | 100% Rickettsia rickettsii^2^ (KF138595),  100%  *Rickettsia bellii*^2^ (KF138596),  100%  *Rickettsia rickettsii*^2^ (ompA) (KF195970) |
| São José do Vale do Rio Preto | *Equus caballus* | *Rhipicephalus microplus* | 1F | omp*A* |  |
|  |  | *Amblyomma sculptum* | 1M | glt*A* and omp*A* | 100% *Rickettsia rickettsii*^2^ (glt*A*) (KF138601) |
|  |  |  | 1F |  | 100% *Rickettsia rickettsii*^2^ *(*glt*A*) (KF138591),  100% *Rickettsia rickettsii*^2^ (ompA) (KF195969) |
|  | *Bos taurus* | *Rhipicephalus microplus* | 2F | glt*A* and omp*A* | 100% *Rickettsia rickettsii*^2^ (glt*A*) (KF138602) |
|  |  |  | 5F | omp*A* |  |
| Barra do Piraí | *Equus caballus* | *Amblyomma sculptum* | 3M | omp*A* |  |
| Paraty | *Canis familiaris* | *Amblyomma ovale* | 2M | omp*A* |  |
|  |  | *Rhipicephalus sanguineus* | 3N | omp*A* |  |
| Rio de Janeiro | *Equus caballus* | *Amblyomma sculptum* | 1N | omp*A* |  |
| Porto Real | *Canis familiaris* | *Amblyomma sculptum* | 1N | omp*A* |  |
|  |  | *Rhipicephalus sanguineus* | 2F | omp*A* |  |
| Nova Iguaçú | *Canis familiaris* | *Rhipicephalus sanguineus* | 5F | omp*A* |  |
|  | Free Living |  | 1N | omp*A* |  |
| Trajano de Moraes | *Canis familiaris* | *Amblyomma sculptum* | 2N | glt*A* | 100% *Rickettsia rickettsii*^2^ (glt*A*) (KF138593) |
| Itaperuna | Free Living | *Amblyomma sculptum* | 22L | glt*A and* omp*A* | 100% *Rickettsia rickettsii*^2^ (glt*A*) (KF138597) |
| Resende | *Bos taurus* | *Rhipicephalus microplus* | 1F | glt*A* |  |
| Itatiaia | Free Living | *Amblyomma sculptum* | 18L | glt*A* |  |
|  |  | *Rhipicephalus sanguineus* | 5N | glt*A* |  |
|  | *Homo sapiens* | *Amblyomma sculptum* | 1N | glt*A* |  |
| Paraiba do Sul | *Equus caballus* | *Dermacentor nitens* | 1F | glt*A* |  |

**L**= Larva, **N**= Nymph, **F**= Female and **M**= Male.

^1^Gehrke, 2010. ^2^ Moura-Martiniano *et al*. 2014.

.**Supplementary Table 1.** Relationship between sequencing *Rickettsia,* host and putative vector in the municipalities of Rio de Janerio State (continuation).

| **Municipalities** | **Host** | **Species** | **Rickettsia detection** | | |
| --- | --- | --- | --- | --- | --- |
|  |  |  | **Specimen** | **Gene amplified** | **Identity sequencing (access GenBank)** |
| Porciúncula | *Free Living* | *Ctenocephalides felis* | 2F | glt*A* and omp*A* | 100% *Ricketsia felis* (glt*A*)^1^ (KF138594) 100% *Rickettsia felis* (omp*A*)^1 (^KF195972) |
| Piraí | *Canis familiaris* | *Ctenocephalides felis* | 1F | glt*A* | 99% *Rickettsia felis*^1^(KF138599) |
|  | *Felis catus* |  | 5F | glt*A* and omp*A* | 100% *Rickettsia felis* (glt*A*-CS2)^1^(KF138592)  100% *Rickettsia felis* (ompA)^1^(KF195971) |
| Petrópolis | *Felis catus* | *Ctenocephalides felis* | 3F | glt*A* | 99% *Rickettsia rickettsii*^2^ (JN400377) |
|  |  |  | 2F |  | 100% *Ricketsia rickettsii*^2^ (JN400366) |
|  | *Canis familiaris* | *Ctenocephalides felis* | 4F | glt*A* | 100% *Rickettsia rickettsii*^2^ (JN400367) |
|  |  |  | 2F |  | 100% *Rickettsia rickettsii*^2^ (JN400378) |
|  |  |  | 1F |  |  |
|  | Free Living | *Ctenocephalides felis* | 1F | glt*A* |  |
|  |  |  | 1F | glt*A* | 100% *Rickettsia rickettsii*^2^ (JN400368) |
| Porto Real | *Felis catus* | *Ctenocephalides felis* | 2F+ 2M | omp*A* |  |
|  |  |  | 1F | glt*A* and omp*A* |  |
| Trajano de Moraes | *Canis familiaris* | *Ctenocephalides canis* | 2F | glt*A* | 100% *Rickettsia rickettsii*^1^ (KF138600) |
| Volta Redonda | *Canis familiaris* | *Ctenocephalides felis* | 1M | glt*A* | 100% *Rickettsia rickettsii*^1^ (KF138598) |
| Nova Iguaçú | *Canis familiaris* | *Ctenocephalides felis* | 3F+ 2M | omp*A* |  |
| Barra do Piraí | *Canis familiaris* | *Ctenocephalides felis* | 4F + 1M | glt*A* and omp*A* |  |
|  |  |  |  | glt*A* |  |

**L**= Larva, **N**= Nymph, **F**= Female and **M**= Male.

^1^Gehrke, 2010. ^2^ Moura-Martiniano *et al*. 2014.
